# Saccadic Eye Movement Abnormalities in Children with Epilepsy

**DOI:** 10.1371/journal.pone.0160508

**Published:** 2016-08-02

**Authors:** Judith Lunn, Tim Donovan, Damien Litchfield, Charlie Lewis, Robert Davies, Trevor Crawford

**Affiliations:** 1 Department of Psychology, Lancaster University, Lancaster, United Kingdom; 2 Centre for Medical Imaging, University of Cumbria, Lancaster, United Kingdom; 3 Department of Psychology, Edge Hill University, Ormskirk, United Kingdom; UMR8194, FRANCE

## Abstract

Childhood onset epilepsy is associated with disrupted developmental integration of sensorimotor and cognitive functions that contribute to persistent neurobehavioural comorbidities. The role of epilepsy and its treatment on the development of functional integration of motor and cognitive domains is unclear. Oculomotor tasks can probe neurophysiological and neurocognitive mechanisms vulnerable to developmental disruptions by epilepsy-related factors. The study involved 26 patients and 48 typically developing children aged 8–18 years old who performed a prosaccade and an antisaccade task. Analyses compared medicated chronic epilepsy patients and unmedicated controlled epilepsy patients to healthy control children on saccade latency, accuracy and dynamics, errors and correction rate, and express saccades. Patients with medicated chronic epilepsy had impaired and more variable processing speed, reduced accuracy, increased peak velocity and a greater number of inhibitory errors, younger unmedicated patients also showed deficits in error monitoring. Deficits were related to reported behavioural problems in patients. Epilepsy factors were significant predictors of oculomotor functions. An earlier age at onset predicted reduced latency of prosaccades and increased express saccades, and the typical relationship between express saccades and inhibitory errors was absent in chronic patients, indicating a persistent reduction in tonic cortical inhibition and aberrant cortical connectivity. In contrast, onset in later childhood predicted altered antisaccade dynamics indicating disrupted neurotransmission in frontoparietal and oculomotor networks with greater demand on inhibitory control. The observed saccadic abnormalities are consistent with a dysmaturation of subcortical-cortical functional connectivity and aberrant neurotransmission. Eye movements could be used to monitor the impact of epilepsy on neurocognitive development and help assess the risk for poor neurobehavioural outcomes.

## Introduction

Childhood onset epilepsies are associated with neurobehavioural comorbidities that can predate the onset of seizures and may persist beyond remission [1]. Research with children with recent onset epilepsies has shown that sensorimotor deficits are a shared abnormality across epilepsy syndromes [2]. Recent analysis of interrelationships between different neurocognitive domains also found a weakened and less well-organized network structure compared to healthy children, with attention and executive function as the two most isolated and poorly integrated domains [3]. Neuroimaging work indicates structural and functional alterations in widespread subcortical and cortical regions in some children with epilepsy, and disruptions to the development of neural network connectivity are causal mechanisms implicated in neurobehavioural comorbidities [4].

The prosaccade and antisaccade task has been used to characterise the neurophysiological and neuroanatomical substrates of fixation and reflexive and voluntary eye movements [5–7]. Neuroimaging work with this task has also mapped the increase in subcortical-cortical connectivity that scaffolds functional integration of oculomotor, attentional and inhibitory control processes from childhood to adulthood [8–11]. This has provided important comparison data for the substantial body of work that has addressed atypical task performance in neurodevelopmental and neuropsychiatric populations [12].

Two prior studies have used eye movement tasks to assess neurocognitive functions in pediatric epilepsy [13,14]. These consistently found increased inhibitory deficits in younger patients that were not clearly attributable to structural brain abnormalities, global developmental delay or epilepsy-related factors. It was suggested that early delay is followed by compensatory mechanisms that normalized functioning by adolescence [14]. In one study, slower processing speed and impaired accuracy were further linked to comorbid attention deficits [13], whereas inhibitory deficits did not discriminate between patients with or without comorbid ADHD. Although epilepsy was assumed to play a role in the observed deficits in performance, neither of the studies reported significant relationships with epilepsy related factors.

Correct antisaccade performance relies on the ability to implement a consistent neural ‘task set’ that is initiated in the prefrontal cortex and orchestrates neural activity in oculomotor midbrain and brainstem structures responsible for fixations and saccades [7]. Errors reflect excessive pre-saccadic activity in subcortical structures that can be gauged from short-latency early saccades [15] or express saccades [16] and, as yet, no study has assessed saccade dynamics in childhood epilepsies. These parameters can probe underlying pathophysiological mechanisms in subcortical structures that may be contributing to weakened functional integration underpinning neurobehavioural comorbidities in this population.

The aims of the present study are to assess previously unexamined eye movement parameters in children with epilepsy, that include processing speed, dynamics, accuracy, error performance and express saccades, and to assess the role of epilepsy factors in oculomotor and neurocognitive development in children at increased risk for poor neurobehavioural outcomes.

## Materials and Methods

### Participants

The study involved a total of 74 children aged 8 to 18 years old, 26 patients and 48 healthy control children. The inclusion criteria for patients were children with epilepsy in mainstream education with presumed genetic or unknown etiology without identifiable structural or metabolic abnormalities. The research program’s recruitment strategy has been previously described [17]. At recruitment to the research program a pediatric neurologist classified patients in accordance with the revised terminology proposed by the International League Against Epilepsy (ILAE) 2005–2009 [18]. Recent classifications of drug resistant epilepsy [19] or resolved epilepsy [20] could not be applied at recruitment to the research program. Therefore the terms ‘chronic’ and ‘controlled’ have been adopted here. Age at epilepsy onset, and duration was derived from medical records at recruitment and parents provided updated information on seizure recency and medications at study participation. In the present sample there were six children (23%) classified with a syndrome (1 with Childhood Absence Epilepsy, 5 with Benign Epilepsy with Centrotemporal Spikes) with the remaining children classified by mode of seizure onset (focal N = 6, generalised N = 8, features of both N = 5, or indeterminate N = 1). Fifteen (57%) of the patients were in receipt of antiepileptic drugs (chronic group) whereas eleven children were unmedicated with four who had never taken antiepileptic drugs (controlled group). Patients’ IQs ranged between 60–121 with eight children in the mild intellectual disability range of 60–80 IQ points. Additional details on clinical characteristics of the patients are provided in S1 Table.

The healthy control (HC) group of typically developing children was recruited via a university research database. A larger number of controls were recruited to improve the reliability of the estimates in the group comparisons, given the large age range included in the study. All 74 children had normal or corrected to normal vision and none had received a diagnosis of a learning disability or a neurodevelopmental disorder. No IQ estimates were collected from controls as this group was recruited from a typical population and would not have matched those patients who had a below average IQ. Participant information is displayed in Table 1.

**Table 1:** Participant Information.

	Controls (n = 48)	Patients (n = 26)	
		Chronic	Controlled
		(n = 15)	(n = 11)
Gender (M: F)	24:24	6:9	5:6
Age (years, SD)	13.1 (2.6)	13.1 (2.4)	12.5 (2.8)
CBCL Attention Problems (SD) ^a^^.^	53.9 (5.3)	63.1 (12.4)	54.9 (6.2)
Borderline or clinical range (%)	2 (4%)	6 (40%)	1 (10%)
IQ (SD)		86.7 (15.9)	93.8 (16.4)
Age at onset (years, SD)		6.5 (2.0)	7.9 (2.6)
Duration (years, SD) ^b^^.^		5.5 (2.8)	2.0 (1.6)
Last known seizure (years, SD) ^c^^.^		0.5 (2.5)	3.2 (1.9)
Present mono / poly therapy (N)		11 / 4	
Prior none / mono / poly therapy (N)		0 / 8 / 7	4 / 6 / 1

^*a*.^ Chronic epilepsy patients had higher reported attention problems than controls (p = 0.033).

^*b*.^ Longer epilepsy duration in patients with chronic epilepsy (p = 0.001).

^*c*.^ More recent last known seizure in patients with chronic epilepsy (p = 0.005).

### Saccadic eye movement task

Eye Movements were captured with an Eyelink 1000 Desktop Mount eye tracker (SR Research) at a monocular sampling rate of 1000 Hz. Children were seated 60 cm from a flat 19-inch LCD monitor (60 Hz refresh rate) with a desktop mounted chin rest. The Experiment Builder program (SR Research) was used to design the task. The task parameters were identical for prosaccades and antisaccades, only the instructions provided to participants varied dependent on the required saccade task.

Trials began with a centrally presented fixation cross that subtended 1° of visual angle. After 1s a green circular 1° sized target was presented in random order at an 8° eccentricity to the left or to the right of the central fixation cross. The fixation cross and target remained on the screen for a period of 1s (an overlap paradigm). On prosaccade trials children were instructed to fixate on the cross until target onset and then to look quickly and accurately toward the target. On antisaccade trials children were told to inhibit a saccade toward the target and to look to the opposite side of the screen. Subsequent to a nine-point calibration procedure and a practice demonstration of correct performance children completed 30 prosaccade trials and 30 antisaccade trials. The trials were presented in two blocks of 15 trials with the instructions repeated after each block. A screen that displayed the eye-traces allowed the experimenter to monitor children’s performance continuously.

### Eye movement data

Criteria for valid saccades were a first horizontal eye movement from the onset of the target with a saccadic reaction time (SRT) between 80 and 1000 ms and with an amplitude equal to or greater than 1° of visual angle. Corrected antisaccade errors were secondary saccades after prosaccade errors with an onset less than 300 ms after error offset and in the direction opposite to the target [21]. Trials were excluded if the eyes were not fixated centrally at target onset, or contained eye blinks or head movements. There was data loss for one HC child on the prosaccade task (technical error) and one patient for the antisaccade task (no valid trials due to noncompliance).

Measures of interest were the reciprocal of SRT (latency or ‘promptness’) that obeys a Gaussian distribution. The mean (μ) and standard deviation (σ) parameters of the main distribution of prosaccades as specified by the LATER (Linear Approach to Threshold with Ergodic Rate) model of reaction time and decision [22] calculated using the software SPIC (available at http://www.cudos.ac.uk/later.html) that produced the LATER parameters for each child by minimisation of the Kolmogorov-Smirnov one-sample statistic. Peak velocity (degrees per second) and gain (saccade amplitude / target amplitude in degrees of visual angle) of correct visually guided prosaccades (PS) and correct antisaccades (AS) and prosaccade errors (PE). As peak velocity is dependent on amplitude a linear regression equation was calculated for each child for each saccade type and used to estimate peak velocity at 8 degrees amplitude. Measures further included the proportion of correct PS in the express range of 80 to 120 milliseconds (ES), and the third parameter in LATER, the standard deviation of the subpopulation of early saccades (early sigma σ_E_) in each child’s prosaccade distribution. The proportion of prosaccade errors committed in the antisaccade task (error rate) and the proportion of errors committed that were subsequently corrected (errors corrected). Too few erroneous saccades were committed in the prosaccade task to permit analysis (n = 9).

### Behavioural Problems

The attention problems scale from The Child Behavior Checklist (CBCL), 6–18 years was used in the study. The scale was measured in T scores with a mean of 50 and SD of 10. A score above 65 was considered to identify clinical problems [23].

### Statistical Analyses

The number of valid trials available for analysis did not differ significantly between the two patient groups and HC but varied between individual children. Two-level random intercept multilevel linear mixed-effects models were used to analyse the measures of reciprocal SRT, peak velocity and gain using the individual trial level data. The subdivision of patients into the chronic and controlled groups explained more variance in performance compared to using only two groups (i.e. patients vs. healthy controls). Therefore group comparisons involved full factorial models with a fixed effect of saccade type (PS, AS, PE), a fixed effect of group (chronic epilepsy, controlled epilepsy, HC) and the interaction term. Participant ID was entered as a random effect. Proportional data on error rates, errors corrected, ES and the LATER early sigma parameter σ_E_, and peak velocity estimated as 8 degrees were analysed with general linear models (GLM). The alpha level in all pairwise group comparisons was Bonferroni adjusted.

Non-linear trends between age and performance were assessed with curve estimation prior to analyses. Age-related effects were analysed by adding chronological age as a fixed effect to models of task performance in patients and HC. In models of epilepsy-related effects (onset, duration, last known seizure) on SRT, peak velocity and gain, patient group and IQ were entered as fixed effects. Although IQ was unrelated to the outcome measures it was included in order to test for epilepsy effects on saccades independent of the relationships between younger onset, increased duration and lower IQ found in patients. Epilepsy factors were entered in a stepwise fashion and results of significant individual fixed effects reported.

To assess the relationships between attention problems and task performance, the scaled scores were entered as a fixed effect into the models after patient group and IQ. The epilepsy variables did not predict attention problems and were excluded from the models. The relationships between proportional data (error rates, errors corrected, ES) early sigma, epilepsy factors and attention problems were analysed with bivariate correlations and multiple linear regression. All statistical analyses were performed in SPSS version 21.0 (IBM Corp., Armonk, NY).

### Ethics

University and National Health Service ethics committees approved the study. All parents provided informed written consent and children informed written assent prior to participation.

## Results

### Group Comparisons

Means (SD) of the saccade measures are reported in Table 2. Results of the mixed-effects models on the saccade SRT and metrics are reported in S2 Table. SRT: There were no significant group differences in the reciprocal SRT of PS, AS or PE (all p = 1.0). There were significant differences in the σ of the PS main distribution (F _1, 70_ = 3.99, p = 0.03, η^2^ = .10). The chronic epilepsy group had a significantly larger σ than controls (p = 0.03). S1 Fig displays a reciprobit plot of the cumulative distributions of PS for the three groups. Peak velocity: The chronic epilepsy group showed higher AS peak velocity than HC (p = 0.02). Gain: The chronic epilepsy group also had higher AS gain compared to HC (p < 0.001) and the controlled epilepsy group (p = 0.03). A general linear model on the estimates of peak velocity at 8 degrees amplitude found no significant effect of saccade type (F _2, 194_ = 0.39, p = 0.68, η^2^ = .004) and no significant effect of group (F _2, 194_ = 0.33, p = 0.72, η^2^ = .003) and no significant interaction (F _4, 194_ = 0.23, p = 0.91, η^2^ = .01). Data were missing for four cases in the AS and four cases in PE analyses as an estimate could not be calculated due to a low number of trials. S3 Table reports the means (SD) of peak velocity at 8 degrees for PS, AS and PE for patient groups and healthy controls.

**Table 2:** Means (SD) of saccade measures in healthy controls and patients.

		Controls (n = 48)	Patients (n = 26)	
			Chronic (n = 15)	Controlled (n = 11)
Reciprocal SRT Hz	PS	6.04 (1.38)	6.03 (1.96)	5.50 (1.78)
	**AS**	**3.56 (1.07)**	**3.43 (1.26)**	**3.49 (0.67)** ^a^^.^
	PE	7.54 (1.78)	7.73 (1.66)	7.01 (2.04)
Peak Velocity	PS	334.6 (79.5)	335.9 (79.8)	344.4 (87.4)
	**AS**	**334.3 (95.7)**	**380.5 (124.1)**	**339.1 (103.5)**
	PE	290.6 (81.3)	310.8 (65.6)	311.1 (93.0)
Gain	PS	1.00 (.22)	.95 (.24)	1.02 (.23)
	**AS**	**1.21 (.50)**	**1.43 (.57)**	**1.22 (.56)**
	PE	.82 (.27)	.83 (.22)	.87 (.27)
Proportion Error Rate		**.36 (.30)**	**.65 (.27)**	**.48 (.32)**
Proportion Errors Corrected		**.90 (.15)**	**.79 (.19)**	**.65 (.26)**
Proportion Express PS		.14 (.16)	.14 (.13)	.08 (.04)
Early Sigma PS σ_E_		4.9 (2.2)	4.9 (2.8)	4.8 (2.0) ^b^^.^

Note: Mu (μ) and sigma (σ) of AS and PE calculated from grouped data. Significant effects are shown in bold and the group comparisons reported in the text.

^*a*.^ Effect is significant only after adjustment for epilepsy duration. PS = prosaccades. AS = antisaccades. PE = prosaccade errors.

^*b*.^ N = 10 as the parameter σ_E_ could not be estimated for one child.

**Fig 1:**
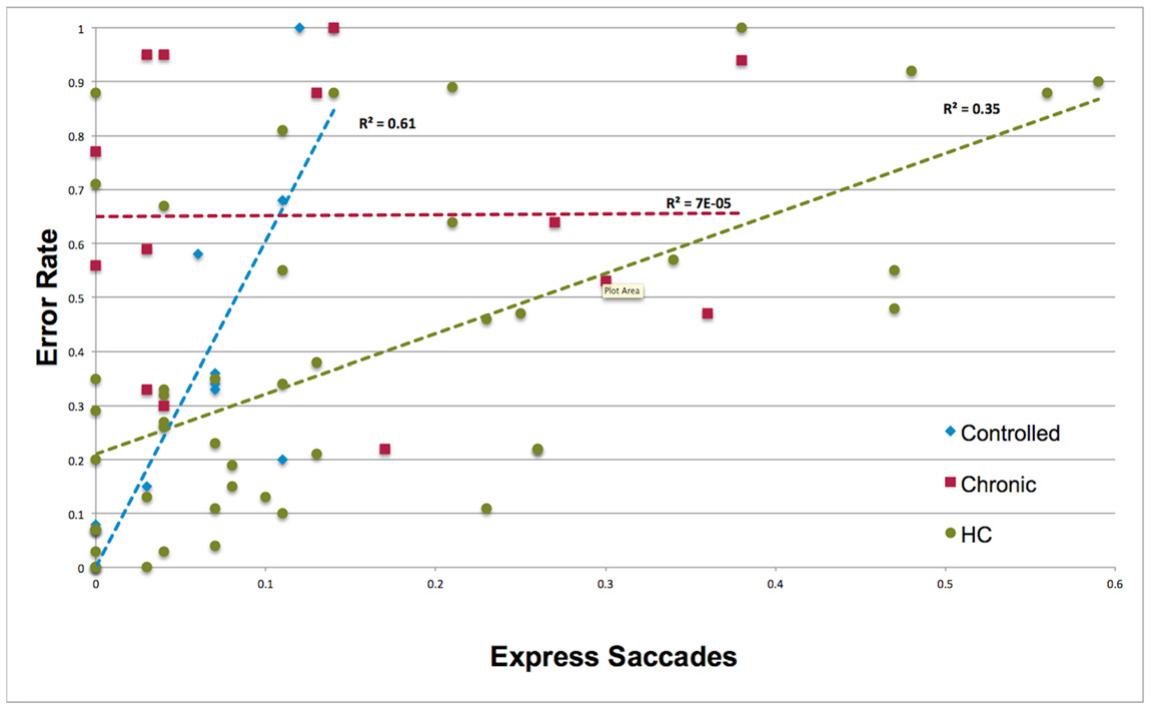
The relationship between express saccade and error rates.

Error rate: The chronic epilepsy group had a higher error rate than HC (F _2, 70_ = 5.16, p = 0.008, η^2^ = .13). Errors corrected: The proportion of errors corrected in the controlled epilepsy group was significantly smaller compared to HC (F _2, 66_ = 9.12, p < 0.001, η^2^ = .22). ES: There were no group differences in the proportion of ES or in σ_E._ In HC and the controlled epilepsy group a higher proportion of ES significantly predicted a higher error rate (Fig 1) and fewer errors corrected (S2 Fig). These relationships were non-significant in patients with chronic epilepsy. No significant relationships were found in the analysis of σ_E._ and error rates.

### Age—related effects

SRT and metrics: Analyses in HC found that SRT of PS, AS and PE significantly decreased with age (all p ≤ 0.04). Peak velocity of AS, PS and PE showed a quadratic relationship with age in HC (all p ≤ 0.03) with a peak at 12–14 years. PE gain reduced linearly with increasing age (p = 0.007). Analyses of peak velocity estimates at 8 degrees amplitude found the quadratic relationship remained for PS (p = 0.003) but was non-significant for AS (p = 0.38) and PE (p = 0.08). No other age-related effects were found. In patients, no age-related effects were found for SRT, raw or estimated peak velocity or gain.

Error rates, errors corrected, ES and σ_E_: In HC there was a marginal reduction in errors with age (F _1, 46_ = 3.41, p = 0.07, η^2^ = .07). There was no significant increase in the proportion of errors corrected (F _1, 46_ = 1.65, p = 0.21, η^2^ = .04). In patients, there was a significant reduction in error rate with age (F _1, 21_ = 12.17, p = 0.002, η_p_^2^ = .37) independent of an effect of IQ (F _1, 21_ = 9.99, p = 0.005, η_p_^2^ = .32). Age also predicted a significant increase in errors corrected (F _1, 21_ = 11.63, p = 0.003, η_p_^2^ = .36) independent of an effect of IQ (F _1, 21_ = 4.49, p = 0.046, η_p_^2^ = .18) and the controlled epilepsy group corrected significantly fewer errors than the chronic epilepsy group (F _1, 22_ = 7.99, p = 0.01, η_p_^2^ = .28). In analyses of ES an effect of age was found only in the controlled epilepsy group (F _1, 9_ = 21.98, p = 0.002, η^2^ = .73) where younger age predicted a higher proportion of ES. No age-related effects were found for σ_E_ in patients or controls.

### Epilepsy—related effects

SRT and metrics: An increase in reciprocal SRT (reduced latency) of PS was significantly predicted by an earlier age at epilepsy onset (F _1, 19.9_ = 5.56, p = 0.029) and longer epilepsy duration (F _1, 20.4_ = 6.98, p = 0.015). An increase in reciprocal SRT of AS was significantly predicted by longer duration (F _1, 15.8_ = 5.53, p = 0.03). After controlling for epilepsy duration, the chronic epilepsy group had significant longer AS latencies than the controlled epilepsy group (F _1, 14.9_ = 4.58, p = 0.05. The negative relationship with duration were driven by patients with a duration of epilepsy > 5 years with faster SRT whom were in receipt of levetiracetam (LEV) (S3 Fig). The σ of the PS main distribution was not significantly predicted by epilepsy developmental variables.

**Fig 2:**
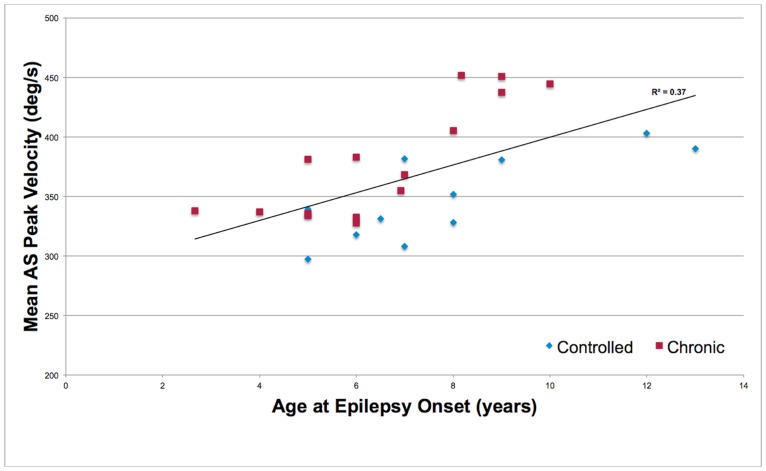
The relationship between age at epilepsy onset and peak velocity of antisaccades in chronic and controlled epilepsy patients.

Analyses of peak velocity found a significant effect of age at epilepsy onset for AS velocity (F _1, 17.8_ = 6.35, p = 0.02). Older age at onset was related to faster AS peak velocity (Fig 2). No significant epilepsy-related effects were found in the analyses of gain. The relationship between age at epilepsy onset and estimated peak velocity at 8 degrees amplitude was non-significant for PS (F _1, 21_ = 3.09, p = 0.09) whereas it was significant for AS (F _1, 15_ = 7.92, p = 0.01) and PE (F _1, 20_ = 8.05, p = 0.01).

**Fig 3:**
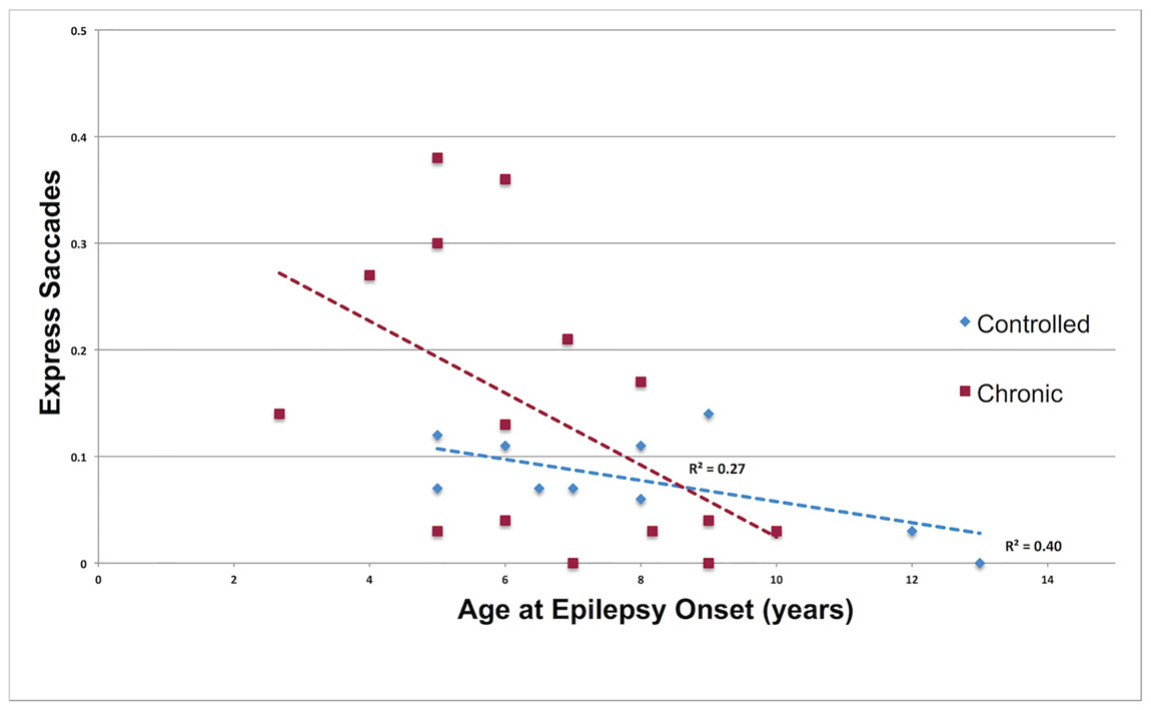
The relationship between age at epilepsy onset and the proportion of express saccades in the prosaccade task.

Error rates, errors corrected, ES and σ_E_: Epilepsy developmental variables did not predict error rates, errors corrected or σ_E_. An increase in ES was significantly related to a younger age at onset (F _1, 21_ = 11.78, p = 0.003, η_p_^2^ = .36) after controlling for age (F _1, 21_ = 5.32, p = 0.03, η_p_^2^ = .20) and IQ (F _1, 21_ = 0.32, p = 0.58, η_p_^2^ = .02) (Fig 3).

### Behavioural Problems

SRT and metrics: Attention problems were related to increased SRT of PS (F _1, 21.3_ = 5.25, p = 0.03) and a trend toward predicting increased latencies of AS (F _1, 18.7_ = 3.12, p = 0.09). Increased attention problems also predicted higher peak velocity of PS (F _1, 21.2_ = 4.57, p = 0.04) and PE (F _1, 20.5_ = 8.28, p = 0.009) and with a trend toward an independent effect in AS (F _1, 22.7_ = 3.34, p = 0.08). No relationship between attention problems and gain were found. The relationship between attention problems and estimated peak velocity at 8 degrees amplitude was non-significant for PS (F _1, 21_ = 2.99, p = 0.10), AS (F _1, 21_ = 1.84, p = 0.20) and PE (F _1, 21_ = 2.97, p = 0.10).

Error rates, errors corrected, ES and σ_E_: Attention problems were related to a higher error rate in a bivariate analysis (Rho = .43, p = 0.03) but in multivariate analysis, the scores did not explain any significant additional variance beyond that explained by age and IQ (R^2^ change = .01, p = 0.44). Attention problems were not significantly related to the proportion of errors corrected, ES or σ_E_.

## Discussion

Children with chronic and controlled epilepsy demonstrated saccadic abnormalities and atypical development of oculomotor and neurocognitive functions. Chronic epilepsy patients had impairments in processing speed, dynamics and accuracy, plus greater inhibitory deficits compared to healthy children. Younger patients with controlled epilepsy also displayed difficulties in error monitoring. Epilepsy-related effects were found to influence saccade parameters and the results point toward atypical developmental integration of basic oculomotor and higher order cognitive functions that contribute to neurobehavioural problems.

### SRT: processing speed

Processing speed deficits with increased cognitive demand emerged in the majority of patients with chronic epilepsy, and attention deficits were related to slowing of volitional saccades, consistent with prior research [13]. Age-related reductions in erroneous and correct saccades were also absent in the patient groups, indicating a maturational disturbance in the efficiency of both reflexive and volitional oculomotor response processing. Contributory factors likely include epilepsy-related disruptions to synaptic pruning and myelination leading to inefficient network transmission [4,13]. The larger σ found in the prosaccade distribution of patients with chronic epilepsy also indicates an increase in response variability. An increase in this parameter has been linked to possible dysregulation of ascending noradrenergic projections to the cerebral cortex [24]. Response variability is also a known characteristic of patients with epilepsy [25] and children with attentional deficits [26].

Consistent with prior evidence of effects of antiepileptic drugs (AEDs) on eye movements [27], patients in receipt of LEV were found to have faster AS processing speed. There is no prior data on the effects of LEV on eye movements in children or adults [27]. This a posteriori finding however does match self-reports of improved psychomotor speed in studies of LEV in children and adults [28], a drug known to have positive and negative stimulating effects [29]. Overall, saccadic reaction times appear sensitive to AED effects in children, but may also be subject to significant variability when used in heterogeneous pediatric groups.

### Peak Velocity and Gain: Dynamics and Accuracy

The faster AS velocities in chronic epilepsy patients indicate excitatory signals initiated in frontal regions at response preparation resulted in relatively greater neural activity in the superior colliculus and paramedian pontine reticular formation circuitry responsible for premotor saccadic velocity commands [30]. Peak velocity is coded from the amplitude of omnipause neuron (OPNs) hyperpolarisations that fire at fixation and the peak in burst firing of saccadic burst neurons (SBNs) that discharge shortly prior and during saccades [31]. Based on neurophysiological studies in primates, atypical increases in saccade peak velocities indicate hyperexcitable or disinhibited saccadic burst generators [32] and possible dysregulation of glycinergic, glutamingergic or GABAergic neurotransmission [30,31,33]. The absence of a relative increase in the visually guided prosaccade task however indicates that a neurophysiological imbalance emerged with increased demand on top-down control.

Increased peak velocity with greater cognitive demand has been previously attributed to neurophysiological arousal modulating oculomotor command signals [34]. Increased arousal stimulates excitatory activity in attention and visuomotor networks [35] and OPNs are proposed to act as specific arousal-related neural modulators on orienting systems [33]. This activity would be coded for in saccadic peak velocity. In patients, attentional deployment and maintenance of the correct AS goal set appears associated with increased arousal that resonates in disinhibited brainstem neural activity resulting in observably faster peak velocity.

The positive relationship between AS raw and estimated peak velocity and age at epilepsy onset showed that elevated velocities persisted in older patients independent of patient group. Onset in later childhood coincides with the maturational increase in cortical to subcortical connectivity, and correct AS performance has greater reliance on prefrontal systems during this transitional period [8–11]. This effect suggests this reliance may persist in those with childhood onset epilepsy. Hyperconnectivity between frontoparietal and motor regions during tasks with high cognitive load has been previously observed in adolescents with epilepsy [36]. Faster peak velocities could conceivably reflect similar mechanisms, marking an imbalance in arousal and hyperexcitability across functional neural networks that emerge during the correct performance on cognitively demanding tasks. This is further supported by the significant relationship between age at onset and increased estimated velocity at 8 degrees that was found for both AS and PE, that are both saccade types produced under conditions of higher cognitive load.

In respect to gain, the hypermetric AS saccades in chronic patients would be predicted from the linear velocity-amplitude relationship at this small target eccentricity [37]. The absence of group differences in the estimated peak velocities at 8 degrees amplitude also indicates the velocity—amplitude relationship was similar for patients and controls. Prosaccade accuracy was unaffected suggesting relatively intact visuospatial processing for visually—guided saccades. Antisaccades however are not controlled online to the same degree as prosaccades due to the lack of a visible target. Top—down control also diminishes feedback use for error correction leading to decreased accuracy and stability [38]. In chronic patients, correct AS performance was at a cost to integrated spatial and motor planning required for the antisaccade vector inversion. Impaired accuracy of both AS and memory-guided saccades has been previously reported [13], yet only impaired accuracy of memory-guided saccades was found to relate to attention deficits. In the present study, the relationship between attention problems and peak velocity was only partially explained when velocity was estimated from children’s main sequence parameters. This suggests the possibility that attention deficits are linked to an impaired spatial working memory component in AS accuracy. It remains for future research to properly delineate the relationships between attention deficits, spatial working memory and motor planning in paediatric epilepsy.

### Errors: Inhibitory Control and Error Monitoring

Consistent with prior studies, chronic epilepsy, lower IQ and younger chronological age were associated with impaired inhibitory control [13,14,39]. Epilepsy developmental factors were not directly related to error rates, whereas the relationships with express saccades were informative of the underlying mechanisms contributing to the inhibitory deficits in patients with chronic and controlled epilepsy.

The pattern of more frequent and variable ES typically observed during early childhood [40,41] was found in patients with an onset within this same developmental period. This suggests epilepsy-related pathophysiology is linked to a persistent increase in pre-saccadic activity in occipital-parietal regions and SC sensorimotor networks [42]. This is further supported by the significant relationship found between earlier age at epilepsy onset and reduced latency of prosaccades. Developmental reductions in ES and errors are also attributable to increased stability in attentional engagement at fixation during the saccade preparatory period [16,43]. The absence of any relationship between express saccades and errors in chronic patients is further evidence of reduced functional connectivity between frontoparietal and subcortical oculomotor networks, and thus a related inability to develop a sustained and consistent inhibitory control task set [10]. Younger controlled epilepsy patients demonstrated rates of express saccades and errors equivalent to similar aged patients with chronic epilepsy but were also less likely to make corrections, explaining the group’s overall lower rate of corrected errors. Parents of younger patients did not report more recent seizures. However three of the four patients with the lowest correction rate were those who had not received pharmacotherapy. Future investigations will need to determine if younger patients have persistent difficulties in error monitoring and if this is related to treatment status.

### Limitations

Standard ophthalmic examination and assessment for neurodevelopment or psychiatric disorders was not implemented in the research program. The failure to collect IQ estimates from the control group resulted in an inability to address the relationship between IQ and error rates by comparing this relationship in patients and healthy controls. The use of only 30 trials per condition is lower than the 120 recommended by an internationally standardized protocol for adults [44]. The lower number of trials resulted in an inability to address antisaccade and error latency distributions using LATER. This could have been more informative on the underlying mechanisms involved in antisaccade decision-making [45] and allow comparison with other neurological patient populations [24]. The low number of trials also resulted in some data loss when performing the linear fit functions for estimated peak velocities. An analysis of trial-by-trial effects may also have provided a more detailed assessment of level of consistency in attentional engagement and response monitoring. However a randomised trial order precluded such analyses. The possibility of continuing seizures in the controlled epilepsy group could not be fully ruled out. Tests of specific AEDs were not a priori and effects of LEV were not found for other eye movement parameters and should be interpreted with caution. The study was unable to address specific epilepsy or seizure types and was limited to addressing effects that emerged despite this heterogeneity.

## Conclusion

Children with epilepsy demonstrated a pattern of saccadic eye movement abnormalities that indicate aberrant development of cortical and subcortical functional connectivity and disrupted neurotransmission. Successful Inhibitory control was achieved at a cost to efficiency, and visuospatial motor planning and it was scaffolded by atypical neurophysiological mechanisms in midbrain and brainstem networks. Age at epilepsy onset appears associated with specific developmental perturbations and this has clear implications for understanding the epilepsy-related mechanisms involved in neurobehavioural problems. Patients and their families would benefit from tests of eye movements in neuropsychological assessment, as these can be clinically informative on aspects of neurocognitive status, disease progression and treatment efficacy.

## Supporting Information

S1 Fig Reciprobit plot of the cumulative distributions of PS in patients and healthy controls. (TIFF)

S2 Fig The relationship between express saccades and proportions of errors corrected in patients and healthy controls. (TIFF)

S3 Fig The relationship between duration and mean antisaccade reciprocal reaction time with labelled cases of patients on LEV. (TIFF)

S1 Table Additional details on clinical characteristics of the patients. (PDF)

S2 Table Statistical results of mixed linear models on saccade task performance. (PDF)

S3 Table Means (SD) of peak velocity at 8 degrees amplitude for patients and controls for PS, AS and PE. (PDF)
