# Supplementary figures and images for: Saccadic Eye Movement Abnormalities in Children with Epilepsy
Source: PLoS One. 2016 Aug 2;11(8):e0160508. doi: 10.1371/journal.pone.0160508 (PMC4970731; doi:10.1371/journal.pone.0160508)

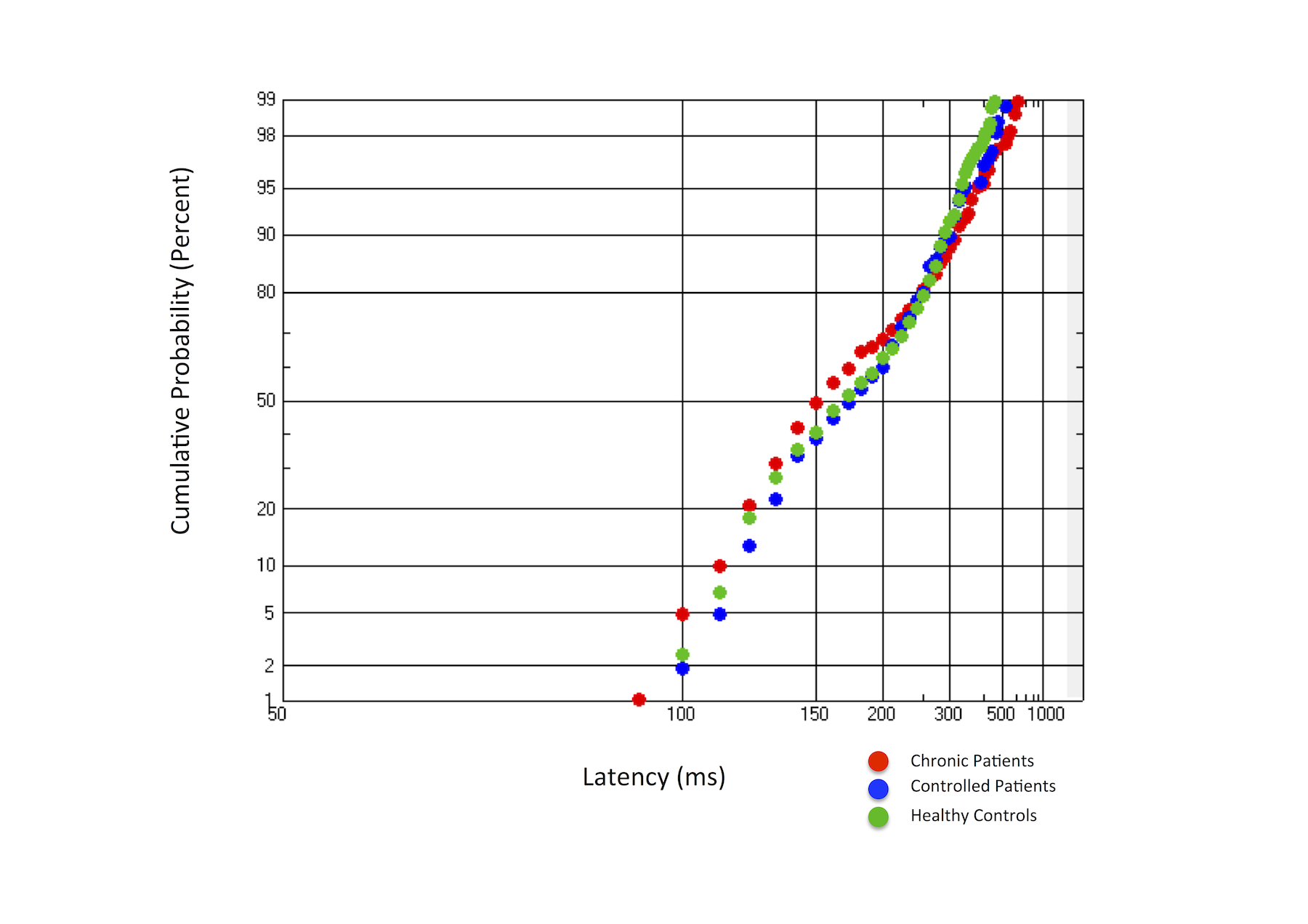

Supplement: S1 Fig — (TIFF) [file pone.0160508.s001.tiff]

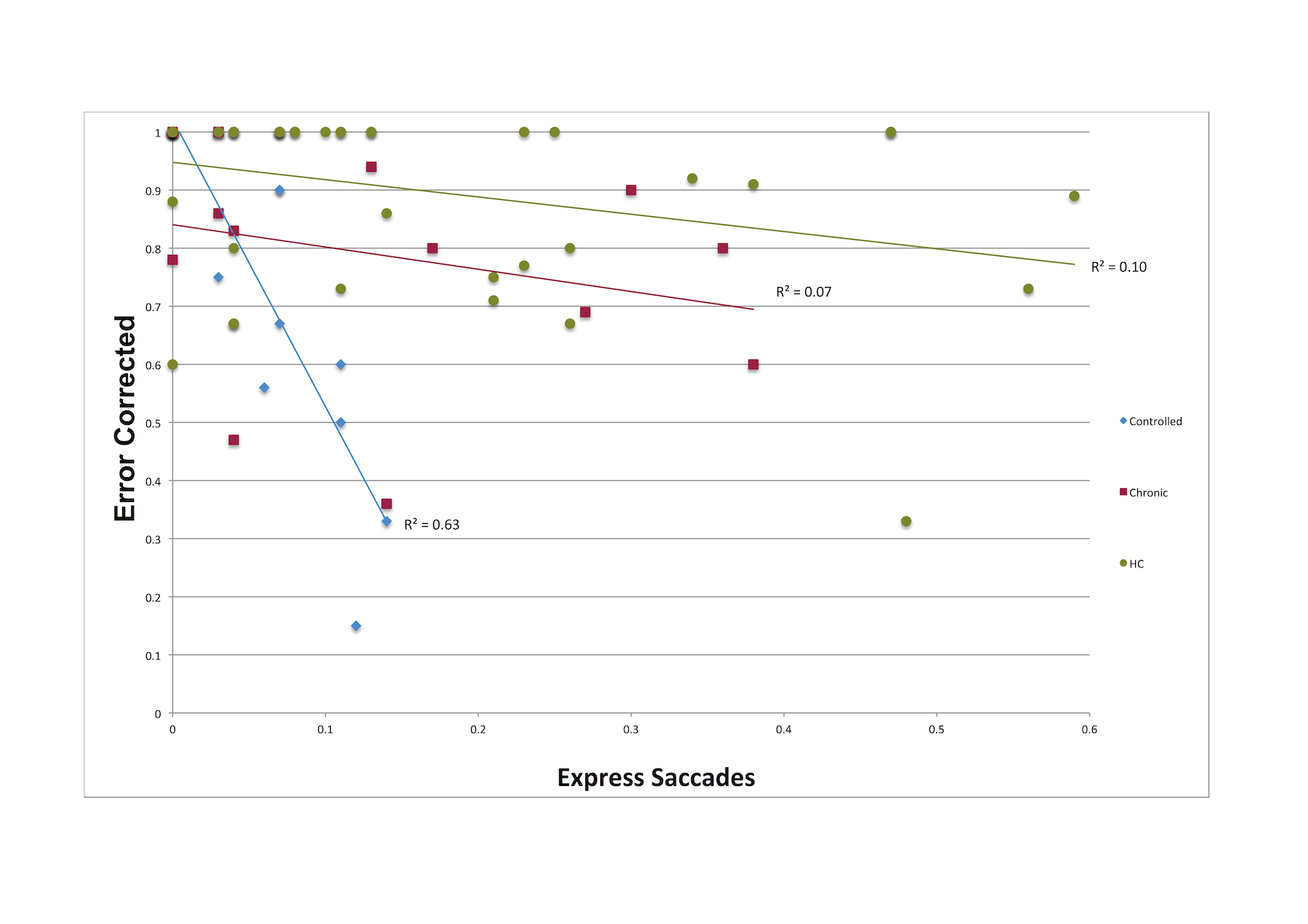

Supplement: S2 Fig — (TIFF) [file pone.0160508.s002.tiff]

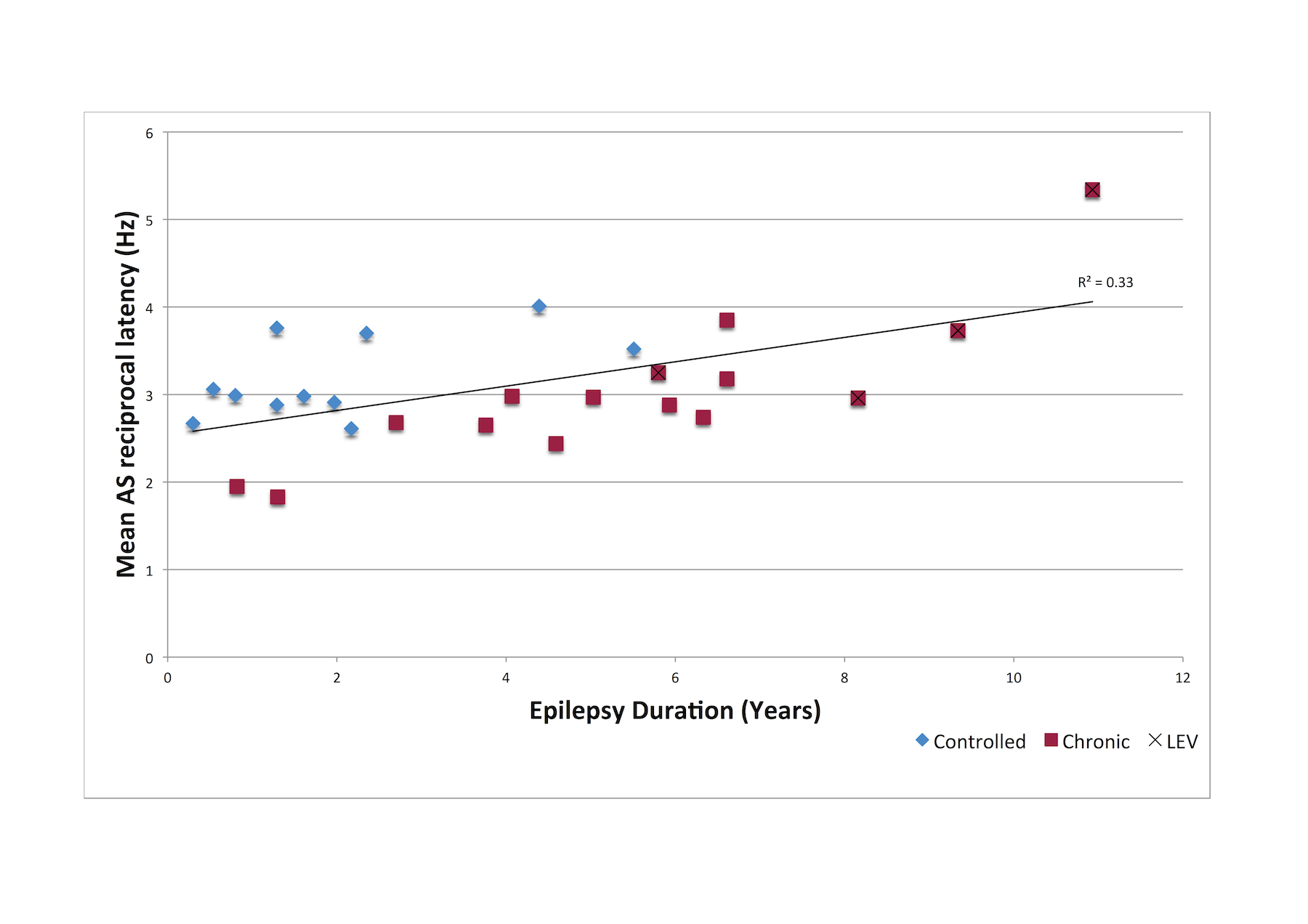

Supplement: S3 Fig — (TIFF) [file pone.0160508.s003.tiff]
