# Supplementary material for: Saccadic Eye Movement Abnormalities in Children with Epilepsy
Source: PLoS One. 2016 Aug 2;11(8):e0160508. doi: 10.1371/journal.pone.0160508 (PMC4970731; doi:10.1371/journal.pone.0160508)
Supplement: S1 Table — (PDF) [file pone.0160508.s004.pdf]

S1 Table. Additional details on clinical characteristics of the patients (N = 26)

	Therapy (n = 15)	No Therapy (n = 11)
Epilepsy Syndromes		
Childhood absence epilepsy		1
Benign epilepsy with centrotemporal spikes	2	3
Non-syndromic epilepsies		
Generalized	4	4
Focal	4	2
Generalized and Focal	4	1
Indeterminate	1	
Seizures in the last 6 months prior to testing	6	1*

* Parental report of a possible seizure in one patient who had never received AEDs.
