# Supplementary material for: Saccadic Eye Movement Abnormalities in Children with Epilepsy
Source: PLoS One. 2016 Aug 2;11(8):e0160508. doi: 10.1371/journal.pone.0160508 (PMC4970731; doi:10.1371/journal.pone.0160508)
Supplement: S2 Table — (PDF) [file pone.0160508.s005.pdf]

S2 Table

Statistical results of mixed linear models on saccade task performance

		Latency	Peak Velocity	Gain
Fixed	Saccade Type	F (2,3627) 543.93, p < 0.001	F (2,3613) 46.89, p < 0.001	F (2,3642) 219.36, p < 0.001
	Group	F (2,73) 0.20, p = 0.75	F (2,73) 1.02, p = 0.36	F (2,77) 1.17, p = 0.32
	Group * Saccade Type	F (4,3626) 3.07, p = 0.015	F (4,3612) 7.22, p < 0.001	F (4,3640) 11.69, p < 0.001
Random	Participant variance	0.78 (0.14), p < 0.001	2725.5 (473.3), p < 0.001	0.018 (.003), p < 0.001
	Residual	2.37 (0.06)	4703.9 (111.1)	0.099 (.002)
