# Supplementary material for: Saccadic Eye Movement Abnormalities in Children with Epilepsy
Source: PLoS One. 2016 Aug 2;11(8):e0160508. doi: 10.1371/journal.pone.0160508 (PMC4970731; doi:10.1371/journal.pone.0160508)
Supplement: S3 Table — (PDF) [file pone.0160508.s006.pdf]

S3 Table. Means (SD) of peak velocity (Deg/Sec) at 8 degrees amplitude for patients and controls for PS, AS and PE.

Saccade Type	Chronic	Controlled	Healthy Controls
PS	369.5 (49.9)	374.2 (51.8)	360.5 (53.1)
AS	354.1 (71.2)	350.4 (65.9)	358.1 (154.3)
PE	376.6 (57.7)	380.8 (44.8)	352.4 (55.9)
